# Accuracy and Reproducibility of Digital Vertical Dimension Increase in Occlusal Splint Design: A Comparative In Vitro Study of Three CAD Software Systems

**DOI:** 10.3390/dj14070402

**Published:** 2026-07-03

**Authors:** Cristian Abad-Coronel, Lesly Abigail Ortiz Miranda, Cristina Adelaida Mariño Arévalo, César A. Paltán, Jorge I. Fajardo, Jaime Larriva, Carolina Encalada

**Affiliations:** 1 Research Group in Digital Dentistry and CAD/CAM Materials, Faculty of Dentistry, Universidad de Cuenca, Cuenca 010107, Ecuador; 2 Faculty of Dentistry, Universidad de Cuenca, Cuenca 010107, Ecuador; 3 New Materials and Transformation Processes Research Group GiMaT, Universidad Politécnica Salesiana, Cuenca 010107, Ecuador

**Keywords:** occlusal splints, vertical dimension of occlusion, CAD/CAM, digital workflow, STL superimposition, OraCheck, finite element analysis, trueness, precision, prosthodontics

## Abstract

**Background**: Digital workflows based on computer-aided design and computer-aided manufacturing (CAD/CAM) enable the modification of the vertical dimension of occlusion (VDO) during occlusal splint design; however, the clinical reproducibility of these digital adjustments compared with conventional methods remains unclear. **Objective**: This in vitro study aimed to evaluate the accuracy and reproducibility of vertical dimension increase achieved with three different dental CAD software systems. **Methods**: A standardized STL dataset was used to design occlusal splints with three CAD software systems: ExoCAD DentalCAD (Exocad GmbH, Darmstadt, Germany), InLab SW 23 (Dentsply Sirona, Bensheim, Germany), and Medit Link (Medit Corp., Seoul, South Korea). A conventional articulator-derived condition served as the comparator. Splints were manufactured using Night Guard Firm 2 Midnight resin (SprintRay Inc., Los Angeles, CA, USA), and the vertical dimension was measured with a digital caliper (Mitutoyo Corp., Kanagawa, Japan). Representative STL superimposition was performed using OraCheck 5.0 (Dentsply Sirona, Bensheim, Germany), and biomechanical behavior was assessed using ANSYS Mechanical 2025 R2 (ANSYS Inc., Canonsburg, PA, USA). Data were analyzed using one-way ANOVA followed by Tukey’s HSD post hoc tests, with the significance level set at α = 0.05. **Results**: The CAD systems showed variable accuracy in reproducing the reference vertical dimension; the Medit Link group yielded values closest to the reference (22.80 mm vs. 22.68 mm), the InLab group demonstrated marked overestimation (26.70 mm), and the ExoCAD group presented intermediate values (24.36 mm). One-way ANOVA confirmed statistically significant differences between the four groups for both sides (*p* < 0.001) with very large effect sizes (η^2^ > 0.98). Three-dimensional superimposition revealed that the InLab splint presented the smallest mean surface deviation from the control (0.36 mm) and the highest proportion of points within 0.1 mm (59%), whereas Medit Link and ExoCAD showed larger mean global distances (0.83 mm and 0.70 mm, respectively). FEA revealed differences in biomechanical behavior, with the highest representative von Mises stress in the Medit Link geometry and the highest representative total deformation in the ExoCAD geometry. **Conclusions**: Digital modification of VDO is feasible, but its accuracy and reproducibility depend on the CAD system used. Careful verification of CAD design parameters against a conventional clinical reference is advisable before manufacturing occlusal splints involving vertical dimension modification, while representative 3D superimposition may provide complementary descriptive information.

## 1. Introduction

Computer-aided design and computer-aided manufacturing (CAD/CAM) technologies have significantly transformed modern dentistry by enabling fully digital workflows for the fabrication of various dental devices. These technologies include both subtractive (milling) and additive (3D printing) manufacturing techniques, offering advantages in dimensional stability, production efficiency, and reproducibility [1,2]. Furthermore, digital workflows contribute to improved patient experience by reducing clinical time and enhancing procedural efficiency [3,4]. Within this context, CAD/CAM systems have been increasingly applied to the design and fabrication of occlusal splints. The workflow typically begins with digital data acquisition through intraoral or extraoral scanners, generating standard tessellation language (STL) files that are processed by CAD software to create customized three-dimensional devices [5].

Occlusal splints are widely used for the protection of dental structures, the management of temporomandibular disorders, and as diagnostic tools in oral rehabilitation [6]. Their primary functions include uniform distribution of occlusal forces, reduction in muscle activity, and stabilization of the maxillomandibular relationship [7]. To achieve these objectives, splints must exhibit specific characteristics such as flat occlusal surfaces, simultaneous contacts, and proper adaptation to the dental arch [8]. They also play a crucial role in the management of reduced VDO, allowing a reversible assessment of changes in VDO before definitive treatment and facilitating the evaluation of functional, esthetic, and phonetic parameters [9]. VDO is defined as the distance between two anatomical reference points; however, there is no consensus regarding the ideal landmarks or a fully reliable method for its determination, and clinical assessment remains multifactorial [10,11].

From a biomechanical perspective, the performance of occlusal splints is influenced by material properties, stress distribution, and deformation under functional and parafunctional loading conditions [12,13]. Finite element analyses have demonstrated that variables such as material composition and thickness can significantly affect stress distribution within dentoalveolar structures [14,15]. Conventional techniques for increasing VDO include the use of anterior jigs, which promote posterior disocclusion and reduce muscular hyperactivity [16]. These methods allow the clinician to determine mandibular position directly in a biological system, providing a clinically validated reference for subsequent treatment planning. Digital workflows have introduced the possibility of modifying mandibular opening virtually within CAD environments, potentially replacing or complementing conventional techniques [17].

Currently, several CAD software platforms are available for splint design, e.g., ExoCAD DentalCAD (GmbH, Darmstadt, Germany), (InLab 23, Dentsply Sirona, Bensheim, Germany), and Medit Link (Medit Corp., Seoul, South Korea), each offering different levels of flexibility, integration, and workflow efficiency [18,19,20]. After the design phase, splints are manufactured using either subtractive or additive techniques. Previous studies have shown that 3D-printed splints may exhibit higher flexibility than milled or conventional devices, while maintaining adequate mechanical performance and clinical effectiveness [21]. Despite these advances, the accuracy and clinical reproducibility of digital VDO modification remain uncertain. Variations in measurement methods, software algorithms, and virtual articulation protocols may affect the final mandibular position, leading to discrepancies between digitally planned and clinically achieved outcomes [22]. A comprehensive accuracy assessment should therefore include both linear (one-dimensional) and three-dimensional surface-based comparisons. The latter, typically performed by best-fit superimposition of the STL meshes, allows the differentiation between trueness—the closeness of the measurement to the true reference value—and precision—the agreement among repeated measurements—as defined by ISO 5725 [23].

To date, limited evidence exists comparing the accuracy and reproducibility of different CAD software systems in achieving controlled increases in VDO relative to conventional reference techniques. Therefore, the aim of this in vitro study was to evaluate the accuracy and reproducibility of vertical dimension increase obtained with three dental CAD software systems for the design of occlusal splints, compared with a conventional articulator-derived reference, by combining linear caliper measurements, three-dimensional STL superimposition with OraCheck 5.0, and finite element analysis. The null hypothesis was that no significant differences exist among the evaluated methods in terms of the accuracy and reproducibility of VDO increase.

## 2. Materials and Methods

### 2.1. Study Design

This study was designed as an in vitro comparative methodological study based on a standardized master digital model. The purpose was to assess the accuracy and reproducibility of vertical dimension increase generated by different CAD software systems under controlled digital conditions (ExoCAD DentalCAD 3.2 Elefsina (exocad GmbH, Darmstadt, Germany), Medit Link (Medit Corp., Seoul, South Korea), and InLab SW 23 (Dentsply Sirona, Bensheim, Germany). A total of 80 occlusal splints/design outputs were evaluated for the linear measurement analysis and distributed into four groups: Control/conventional articulator-derived comparator group (*n* = 20), ExoCAD group (*n* = 20), InLab group (*n* = 20), and Medit Link group (*n* = 20). The three-dimensional STL superimposition analysis was performed on representative splint pairs and was interpreted descriptively. The sample size was determined based on the primary outcome variable, defined as the linear deviation from the primary reference vertical dimension value. Considering the in vitro comparative design, four experimental groups, and the expected large differences among CAD workflows under standardized conditions, a minimum of 20 splints/design outputs per group was selected for the primary linear measurement analysis. This provided a total sample size of 80 specimens/design outputs. A priori power analysis for one-way ANOVA was considered using α = 0.05 and 80% power. The representative STL superimposition analysis was not included in the power calculation because it was performed only as an exploratory descriptive assessment.

### 2.2. Digital Dataset and Reference Model

A standardized master digital model was used as the source dataset to ensure identical anatomical geometry and occlusal relationships across all experimental conditions. Two sets of virtual models in STL format were generated: one in maximum intercuspation (MI) and another with a pre-established mandibular opening obtained after the use of an anterior jig. The STL file with the increased mandibular opening was used as the digital source for reproducing the predetermined vertical dimension; however, it was not considered the numerical reference value for the statistical analysis.

For clarity, the primary linear reference value in this study was defined as the vertical dimension measured directly with a digital caliper on the articulator-mounted physical model at the predetermined increased vertical dimension. This reference corresponded to 22.68 mm on the right side and 21.10 mm on the left side. These values were used as the baseline reference values for calculating linear deviations, or trueness, in all experimental groups.

**Figure 1:**
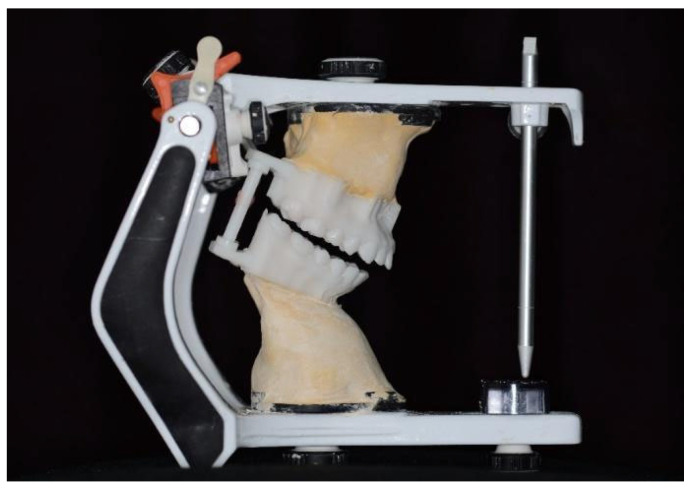
Maxillary and mandibular models mounted on a semi-adjustable articulator (Bioart A7 Plus, Bio-Art Ltd.a, Brazil) at a predetermined increased vertical dimension.

To generate the conventional comparator condition, a typodont model was 3D-printed (Dental Model Stone, SprintRay, Los Angeles, CA, USA), post-processed in a washing and curing unit (SprintRay ProWash, Los Angeles, California, USA/Procure), and mounted on a semi-adjustable articulator (Bioart A7 Plus, Bio-Art Ltd.a, São Paulo, Brazil) at the predetermined increased vertical dimension derived from the STL model with mandibular opening (Figure 1). Splints produced from this articulator-based workflow constituted the Control group. Therefore, the Control group was not treated as the mathematical zero-reference itself, but as a conventional/articulator-derived comparator group measured under the same conditions as the CAD-designed splints.

For each CAD software system, twenty repeated design outputs were generated from the same STL dataset (*n* = 20 per group), allowing the evaluation of both accuracy and reproducibility while isolating the effect of the CAD design workflow. Linear measurements were obtained with a digital caliper (Mitutoyo Corp., Kanagawa, Japan; resolution 0.01 mm; accuracy ±0.02 mm) at reproducible anatomical reference points corresponding to the incisal zeniths. Each measurement was performed three times and averaged to minimize measurement error. For each group, the final vertical dimension value was calculated as the mean of all measurements obtained from the repeated splint designs. The deviation of each group from the primary linear reference value was used to express trueness.

### 2.3. CAD Design Workflow and Manufacturing Process

**Figure 2:**
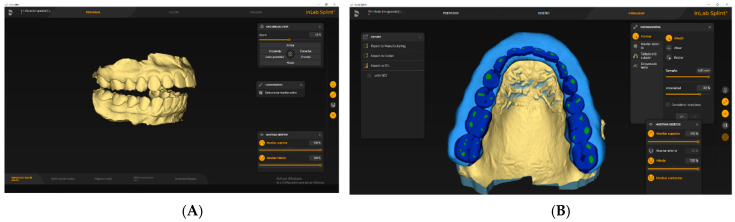
(**A**) STL model with predefined mandibular opening generated in InLab software for occlusal splint design. (**B**) Occlusal contact points identified on the splint surface.

Three study groups were defined according to the CAD software system used: Group 1 (ExoCAD), Group 2 (InLab), and Group 3 (Medit Link). The STL models in maximum intercuspation were imported into each software environment. Using the native splint design tools, mandibular opening was digitally modified to a standardized value of 8.4 mm, corresponding to the maximum opening required to achieve the reference vertical dimension (22.68 mm). For each CAD system, twenty repeated design outputs were generated from the same STL dataset (*n* = 20 per group). Design parameters, including occlusal contact distribution and splint morphology, were maintained as consistently as possible across platforms; nevertheless, due to differences in internal algorithms and occlusal management protocols, variations in the resulting designs were expected. The control splint was designed using a CAD software platform (InLab SW 23, Dentsply Sirona, Bensheim, Germany) and subsequently exported for manufacturing (Figure 2). Although InLab SW 23 was used to generate the control splint from the articulator-derived reference condition, this control workflow was conceptually distinct from the InLab experimental group. The Control group represented the conventional/articulator-derived comparator condition, whereas the InLab group represented repeated CAD design outputs generated from the standardized digital workflow for comparison with the other CAD systems.

**Figure 3:**
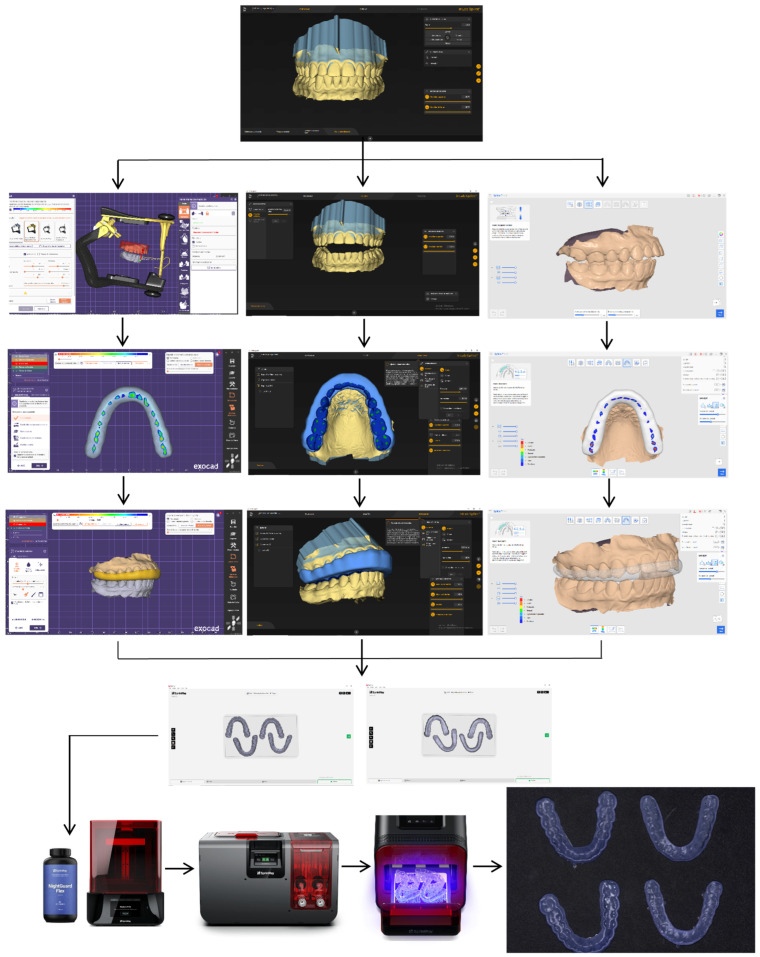
Representative CAD design and additive manufacturing workflow used for the occlusal splints.

All splints were exported and nested with dedicated printing software (RayWare, SprintRay, Los Angeles, CA, USA) and manufactured with a 3D printing system using a splint-specific resin (Night Guard Firm 2 Midnight, SprintRay, Los Angeles, CA, USA) at 100 µm resolution. Post-processing comprised a two-stage washing cycle, drying, and final polymerization in a combined unit (SprintRay ProWash/Procure, SprintRay, Los Angeles, CA, USA) for 4 min. Representative views of the digital design and manufacturing workflow are shown in Figure 3.

### 2.4. Measurement of Vertical Dimension

Each splint was positioned on the articulated models, starting from maximum intercuspation, to record the vertical dimension achieved for each group. Linear measurements were obtained with the digital caliper described in Section 2.2, taken at reproducible anatomical reference points corresponding to the incisal zeniths of the maxillary and mandibular central incisors. This method was selected to provide a simple and clinically interpretable assessment of vertical dimension changes. Each measurement was performed three times and averaged. For each group, measurements were obtained from twenty repeated design outputs per group, and the final vertical dimension value was calculated as the mean of all recorded measurements. All measurements were expressed in millimeters (mm) and compared across experimental groups to determine the accuracy of vertical dimension increase. The primary outcome variable was trueness, defined as the deviation of each measured value from the primary linear reference value.

### 2.5. Free-Body Diagram and Finite Element Boundary Conditions

**Figure 4:**
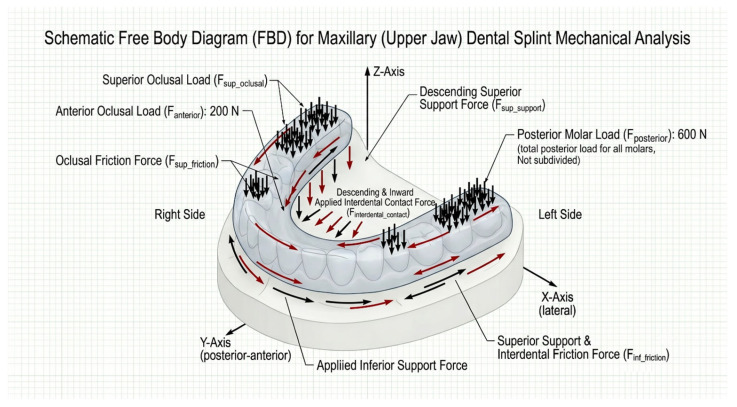
Free-body diagram and finite element boundary conditions used for the occlusal splint simulation. Note: Image generated using artificial intelligence for illustrative purposes.

To improve the reproducibility and interpretation of the finite element model, a free-body diagram was included to illustrate the applied loads, contact formulation, and boundary conditions. In this diagram, the occlusal splint was isolated as the deformable body of interest, while the teeth and supporting bone were represented as rigid support structures. The basal region of the dental/supporting structure was constrained using a fixed-support condition, preventing rigid body motion during loading. The tooth-splint interface was represented as a nonlinear frictional contact, and the occlusal loads were applied as distributed compressive forces over predefined contact regions rather than as point loads (Figure 4).

### 2.6. Finite Element Analysis

**Figure 5:**
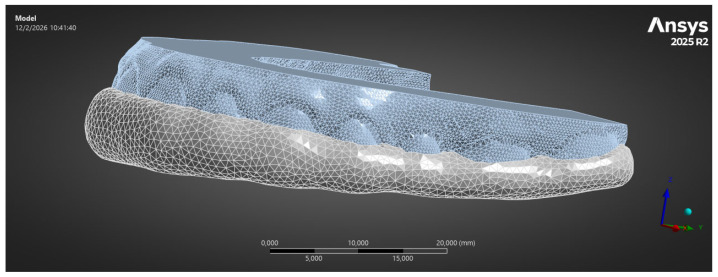
Finite element model showing the geometric assembly of the occlusal splint and the supporting structures used for simulation.

Finite element analysis (FEA) was performed using ANSYS Mechanical 2025 R2 (ANSYS Inc., Canonsburg, PA, USA) to compare the biomechanical response of the four occlusal splint geometries under identical standardized computational conditions. The analysis was designed as a comparative in silico assessment rather than as a definitive prediction of long-term clinical performance (Figure 5).

The geometric assembly included three structural domains: the occlusal splint, the teeth, and the supporting bone. The teeth and bone were modelled as rigid bodies, whereas the occlusal splint was modelled as the only deformable structure. This modelling strategy was selected to isolate the effect of CAD-derived geometric variation on the mechanical response of the splint, avoiding additional confounding effects associated with periodontal, dental, or osseous deformation.

The splint material was modelled as homogeneous, isotropic, and linear-elastic. The assigned mechanical properties were Young’s modulus E = 2.452 GPa and Poisson’s ratio ν = 0.35. These values were selected from previously published finite element studies of rigid PMMA-based and methacrylate-based occlusal splint materials and are consistent with the expected range reported for rigid post-cured splint resins. The material used for manufacturing was Night Guard Firm 2 Midnight (SprintRay Inc., Los Angeles, CA, USA).

The interaction between the intaglio surface of the splint and the dental structures was defined using a nonlinear frictional contact formulation. A friction coefficient of μ = 0.12 was assigned to represent the interface between a rigid photopolymerizable resin and dental enamel under lubricated oral conditions. This formulation allowed localized separation and tangential micromovement when mechanically permitted by the contact state, avoiding the unrealistic assumption of perfect bonding between the splint and the teeth.

A fixed support was applied at the basal region of the dental/supporting structure. In ANSYS, this condition restricted translational displacement in the X, Y, and Z directions, preventing rigid body motion of the assembly during occlusal loading. The nonlinear contact problem was solved using the Newton-Raphson iterative scheme implemented in ANSYS Mechanical, with large deformation enabled.

Occlusal loading was applied as distributed vertical forces over predefined contact areas. A total posterior load of 600 N was distributed over the premolar contact regions, corresponding to approximately 300 N per side, and an additional anterior load of 200 N was distributed over the anterior contact region. Thus, the total applied load was 800 N. This loading scheme was selected to simulate a severe parafunctional loading scenario, such as maximum clenching or bruxism, and to compare the structural response of the four CAD-derived geometries under a standardized worst-case condition. No oblique or lateral loading components were included.

A steady-state oral temperature of 37 degrees C was incorporated into the computational model. The resin was assigned a coefficient of thermal expansion of 80 × 10^−6^ K^−1^, with a reference temperature of 20 degrees C and a final temperature of 37 degrees C. This thermal condition was included to represent the oral environment and the potential thermally induced pre-strain of the splint material. Standard Earth gravity was also included as a constant body force; however, its mechanical contribution was considered negligible compared with the magnitude of the applied occlusal loads.

The models were discretized using higher-order quadratic tetrahedral elements. A global element size of 0.8 mm was used, with local mesh refinement to 0.3 mm in occlusal contact areas, cusp-related regions, borders, and geometrically complex or thin regions of the splint. Mesh quality was evaluated using aspect ratio and skewness. The final meshes contained 145,230 nodes and 88,410 elements for the Control geometry, 152,110 nodes and 92,305 elements for the InLab geometry, 148,890 nodes and 90,150 elements for the ExoCAD geometry, and 150,560 nodes and 91,200 elements for the Medit Link geometry.

A mesh convergence analysis was performed using the maximum equivalent von Mises stress as the target variable. Four refinement levels were evaluated, with element sizes of 1.5 mm, 0.8 mm, 0.5 mm, and 0.3 mm. Convergence was considered achieved when the variation in maximum von Mises stress between two successive refinements was below 5%. The final variation between the fine and optimal meshes was 0.2%, confirming mesh independence for the comparative analysis.

**Figure 6:**
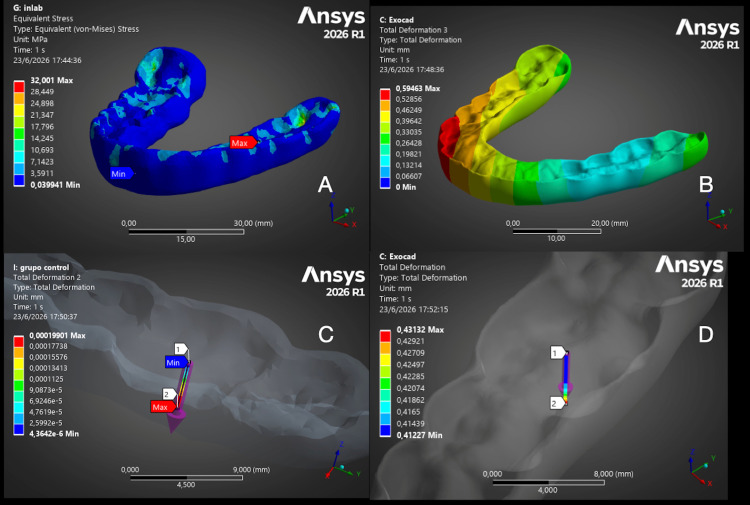
FEA outputs showing von Mises stress, total deformation, and predefined premolar deformation paths.

The primary outcome variables were equivalent von Mises stress and total deformation. In addition to global stress and deformation values, local deformation was evaluated along predefined paths across the splint thickness in the right and left premolar regions. For each path, the minimum deformation, maximum deformation, and path deformation range were reported. The deformation range was calculated as the difference between the maximum and minimum deformation values recorded along the same path (Figure 6). The simulation was deliberately framed as a static analysis with linear-elastic isotropic material behaviour and a single, geometrically standardized loading scenario; cyclic loading, viscoelasticity, fatigue, aging, and other time-dependent phenomena were not modelled. This decision was made to keep all four geometries strictly comparable under identical boundary conditions and to isolate the effect of CAD-induced geometric variability, which was the variable of interest of the present in vitro study [12,13,14,15].

Standard Earth gravity was included as a constant body force for completeness of the computational setup; however, its mechanical contribution was considered negligible compared with the magnitude of the applied occlusal loads.

### 2.7. Path Analysis Methodology

Local deformation across the splint thickness was further evaluated using predefined path analyses in the right and left premolar regions. For each path, the minimum deformation, maximum deformation, and path deformation range (∆ Def_path) were extracted. The path deformation range was calculated as the difference between the maximum and minimum deformation values recorded along the same path:

ΔDef_path = Def_max − Def_min

This parameter was used to describe the local deformation gradient across the splint thickness and to avoid misinterpretation of isolated nodal values.

### 2.8. STL Superimposition Analysis

To complement the linear caliper-based assessment of vertical dimension and to provide a complementary three-dimensional descriptive assessment of the geometric agreement between the CAD-designed splints and the control, a surface-based superimposition analysis was performed using OraCheck 5.0 software (Dentsply Sirona, Bensheim, Germany) [24]. This software is designed for the comparative evaluation of intraoral and extraoral STL datasets and computes point-to-surface distances after a best-fit alignment based on the iterative closest point (ICP) algorithm. Because the three-dimensional superimposition analysis was performed on one representative splint per CAD system per comparison, these results should be interpreted exclusively as exploratory and descriptive surface-based observations. They were not used for inferential statistical comparisons and were not considered confirmatory evidence of differences among CAD systems.

Owing to the computational cost of full-mesh comparison, the three-dimensional superimposition was performed on representative samples randomly selected from each CAD group (one splint per CAD system per pairwise comparison), while keeping the linear caliper analysis on the complete sample (*n* = 20 per group). For this reason, the OraCheck 5.0 surface-based outputs reported in Section 3.7 are interpreted as descriptive indicators of geometric agreement between CAD systems and the control, rather than as inferential estimators; no parametric or bootstrap confidence intervals were computed for the 3D superimposition because the sample size per comparison was *n* = 1. For each comparison, two STL files were imported into OraCheck 5.0: one corresponding to the splint designed with each CAD software system (ExoCAD, InLab, or Medit Link) and the other corresponding to the control splint generated from the conventional articulator-based reference. After automatic best-fit superimposition, signed and unsigned point-to-surface distances were computed across the entire occlusal and intaglio surfaces of the splint. Areas exhibiting non-physiological deviations greater than the predefined cut-off (typically associated with post-processing artefacts) were automatically flagged by the software and reported as “discarded area”.

Six pairwise comparisons were carried out: ExoCAD vs. Control, Medit Link vs. Control, InLab vs. Control, ExoCAD vs. Medit Link, ExoCAD vs. InLab, and Medit Link vs. InLab. For each comparison, the following parameters were extracted: (i) average absolute distance (mm) across the superimposed surfaces, and (ii) distribution of point-to-surface deviations across four predefined intervals (0–0.1 mm, 0.1–0.5 mm, 0.5–1.0 mm and >1.0 mm), plus the percentage of discarded area. Color-coded deviation maps were generated to visually represent regions of agreement and discrepancy. This three-dimensional analysis was performed in addition to—and not as a replacement for—the linear caliper measurements to combine clinically interpretable linear data with surface-based information that integrates the entire geometry of the splint.

### 2.9. Statistical Analysis

Statistical analyses were carried out at a significance level of α = 0.05 using a dedicated statistical software package (SPSS, IBM Corp., Armonk, NY, USA). The normality of the data distribution was assessed using the Shapiro–Wilk test, and the homogeneity of variances was verified with Levene’s test based on the median. Depending on data distribution and variance behaviour, differences between groups were analysed using one-way analysis of variance (ANOVA) followed by Tukey’s HSD post hoc tests when normality and homoscedasticity were satisfied; Welch’s heteroscedastic ANOVA followed by Games–Howell post hoc tests when normality was met but variances were unequal; or the Kruskal–Wallis test with Bonferroni-adjusted Mann–Whitney U post hoc comparisons for non-normally distributed data. Effect sizes were calculated to quantify the magnitude of group differences: eta-squared (η^2^) and omega-squared (ω^2^) for the omnibus test, and Cohen’s d for pairwise comparisons [25]. Trueness was expressed as the deviation of each measurement from the baseline reference value, in agreement with the ISO 5725 framework [23]. Continuous variables are presented as mean ± standard deviation (SD) with 95% confidence intervals (CI). OraCheck-derived superimposition data are presented descriptively as mean absolute distances and as percentages within predefined deviation intervals.

## 3. Results

### 3.1. Linear Measurements of Vertical Dimension

Baseline linear measurements obtained from the control group with the digital caliper in maximum intercuspation (MI) and at the increased vertical dimension are presented in Table 1.

### 3.2. Comparison of Vertical Dimension Achieved by Each Splint

**Figure 7:**
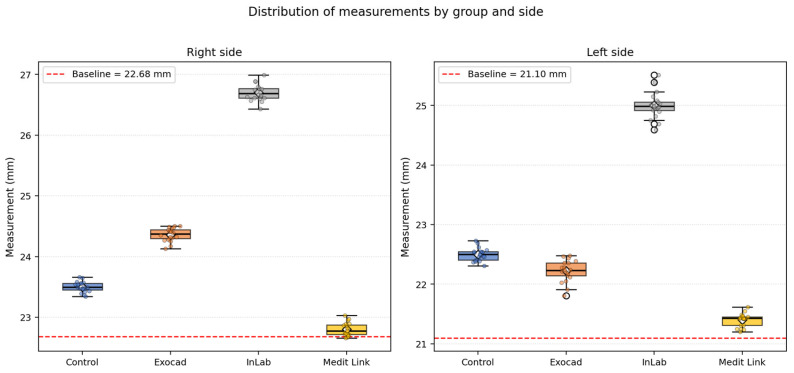
Box-and-whisker plots of the measurements per group for the right and left sides. Boxes denote the interquartile range; horizontal lines mark the medians, white diamonds the means, whiskers extend to 1.5 × IQR, and individual data points are overlaid. The dashed red line indicates the baseline reference value.

**Figure 8:**
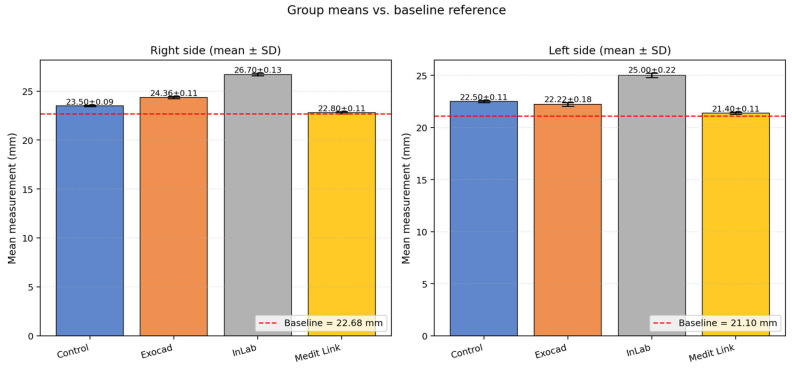
Mean ± SD measurement per group for the right and left sides. The dashed red line represents the baseline reference value used to calculate trueness.

**Figure 9:**
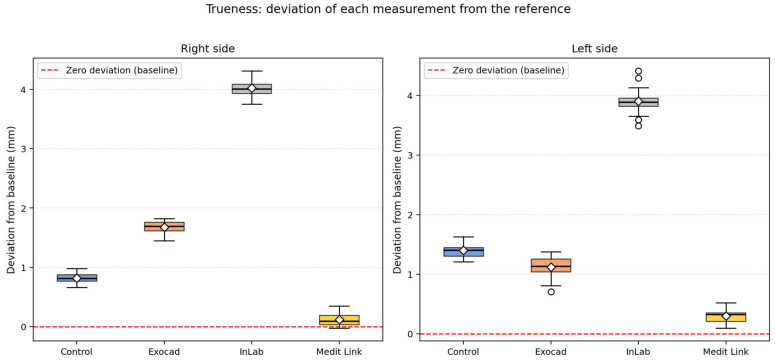
Trueness expressed as the per-measurement deviation from the baseline reference. The horizontal red line at 0 mm indicates perfect agreement with the reference; positive values represent overestimation, negative values represent underestimation.

Table 2 summarises the descriptive statistics of the right and left measurements obtained for each group (*n* = 20 per group). Within-group standard deviations ranged from 0.087 to 0.132 mm on the right side and from 0.107 to 0.215 mm on the left side. The distribution and dispersion of measurements across groups are illustrated in Figure 7. The Control group showed the narrowest dispersion, whereas the InLab group showed the largest dispersion on both sides. Group mean values and variability are presented in Figure 8. The largest absolute deviation from the baseline reference (right = 22.68 mm; left = 21.10 mm) was observed in the InLab group, while the Medit Link group produced the values closest to the reference for both sides. Trueness values expressed as deviation from the baseline reference are illustrated in Figure 9.

The Shapiro–Wilk test indicated that the measurements were normally distributed in all groups for both sides (*p* > 0.05; Table 3). Levene’s test based on the median yielded the following results: right side, W = 1.097, *p* = 0.356 (variances homogeneous); left side, W = 2.073, *p* = 0.111 (variances homogeneous). Therefore, the assumptions for parametric testing were met and one-way ANOVA followed by Tukey’s HSD post hoc comparisons was applied for both sides.

The one-way ANOVA showed statistically significant differences between groups for both the right side (F(3, 76) = 4821.82, *p* < 0.001, η^2^ = 0.995, ω^2^ = 0.994) and the left side (F(3, 76) = 1904.68, *p* < 0.001, η^2^ = 0.987, ω^2^ = 0.986); group membership accounted for more than 98% of the total variance in measurements (Table 4).

Tukey’s HSD post hoc comparisons (Table 5) showed that all pairwise between-group differences were statistically significant (*p* < 0.001) for both sides. The InLab group differed from all other groups, with the largest mean differences and the largest effect sizes (|d| > 14 in every comparison). The Medit Link group showed the smallest absolute deviation from the baseline reference and was the closest to the Control group, although the difference still reached statistical significance.

### 3.3. Stress Distribution Analysis (FEA)

Differences in stress distribution were observed among the evaluated geometries (Table 6). The Medit Link geometry showed the highest von Mises stress range (40.47 MPa), whereas the Control geometry showed the lowest stress range and maximum stress values. The InLab and ExoCAD geometries showed intermediate stress ranges.

### 3.4. Total Deformation Analysis

The Control geometry showed the lowest deformation range and maximum total deformation values. The ExoCAD geometry showed the highest global deformation range (0.0594 mm), whereas InLab and Medit Link showed intermediate deformation ranges (Table 7).

### 3.5. Deformation Analysis Across Premolar Regions

Path-based deformation analysis (Table 8) showed differences in local mechanical behavior between geometries. The control group presented minimal representative deformation in both premolar regions (right: 0.000195 mm; left: 0.000188 mm). The InLab group showed increased representative deformation values (right: 0.019537 mm; left: 0.011030 mm). ExoCAD and Medit Link also exhibited higher representative deformation values than the control, with side-to-side variability.

### 3.6. Summary of Biomechanical Behavior

Under identical loading, material, contact, boundary, thermal, and mesh conditions, the four occlusal splint geometries exhibited different stress and deformation patterns. The Medit Link geometry showed the highest equivalent von Mises stress values, while the ExoCAD geometry showed the highest global deformation values. The Control geometry consistently presented the lowest stress and deformation values, indicating a more homogeneous mechanical response under the standardized simulation conditions.

Path-based deformation analysis was performed across the splint thickness in the right and left premolar regions. For each path, minimum deformation, maximum deformation, and path deformation range (ΔDef_path) were reported. The path deformation range was calculated as the difference between the maximum and minimum deformation values recorded along the same path:

ΔDef_path = Def_max − Def_min

**Figure 10:**
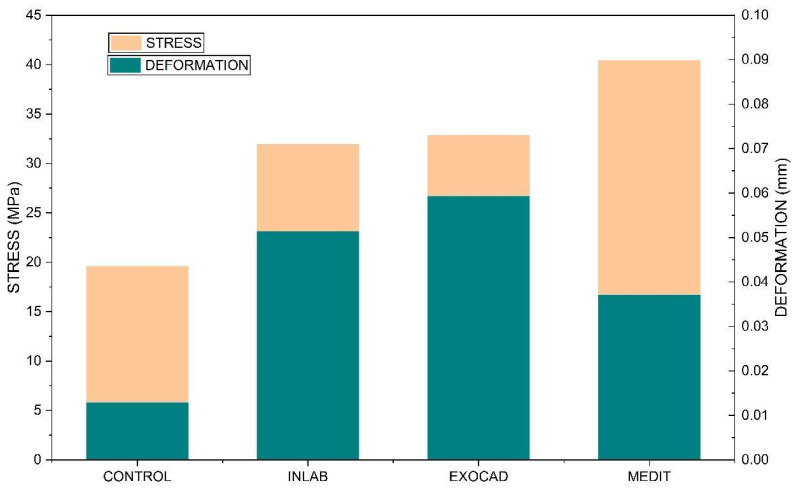
Comparative analysis of von Mises stress (MPa) and total deformation (mm) for the four evaluated geometries (Control, InLab, ExoCAD, and Medit Link).

The Control geometry showed the lowest deformation ranges in both premolar paths, with ΔDef_path values of 0.000195 mm on the right side and 0.000188 mm on the left side. The InLab geometry showed increased local deformation ranges of 0.019537 mm on the right side and 0.011030 mm on the left side. The ExoCAD geometry presented deformation ranges of 0.019050 mm and 0.015860 mm in the right and left premolar paths, respectively. The Medit Link geometry showed deformation ranges of 0.022374 mm on the right side and 0.015860 mm on the left side (Figure 10).

**Figure 11:**
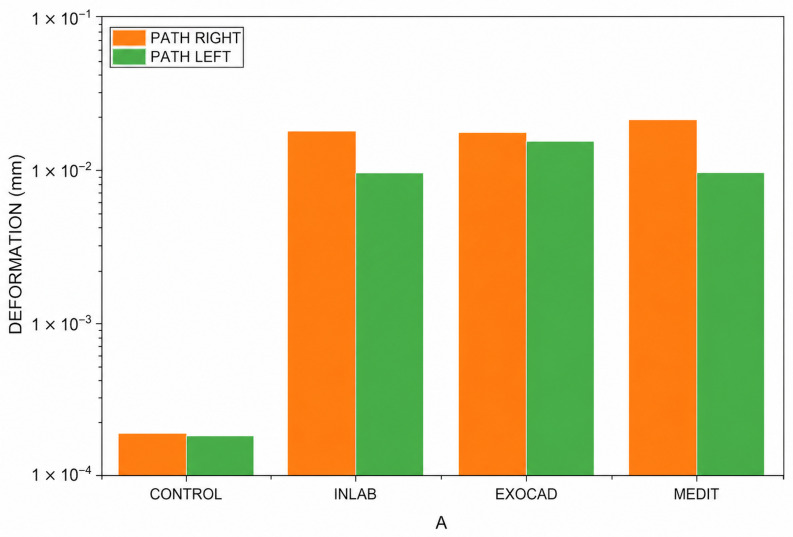
Comparative analysis of total deformation (mm) along predefined right and left premolar paths across splint thickness for the four evaluated geometries.

These findings indicate that CAD-derived geometric differences influenced the local deformation behavior of the splints. While the Control geometry showed minimal deformation gradients across the evaluated paths, the CAD-designed geometries exhibited higher deformation ranges, suggesting greater local deformation gradients in the premolar regions under the same standardized loading conditions (Figure 11).

Figure 11 illustrates the path-based deformation ranges across the right and left premolar regions for the four evaluated geometries.

### 3.7. Descriptive Three-Dimensional Surface Assessment (OraCheck Superimposition)

**Figure 12:**
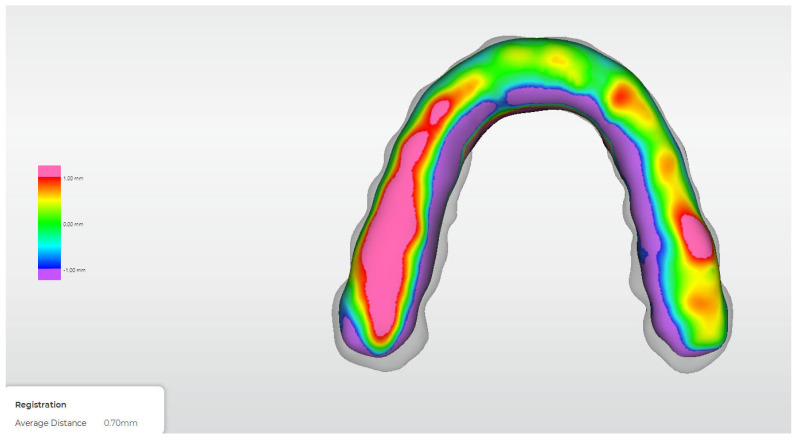
OraCheck 5.0 colour-coded deviation map of the ExoCAD splint vs. Control superimposition.

**Figure 13:**
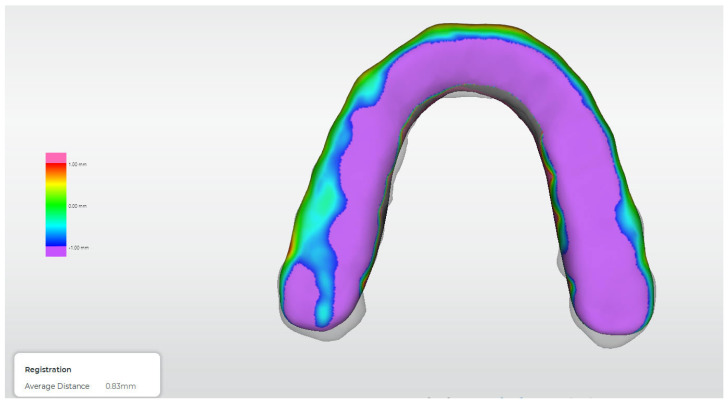
OraCheck 5.0 colour-coded deviation map of the Medit Link splint vs. Control superimposition.

**Figure 14:**
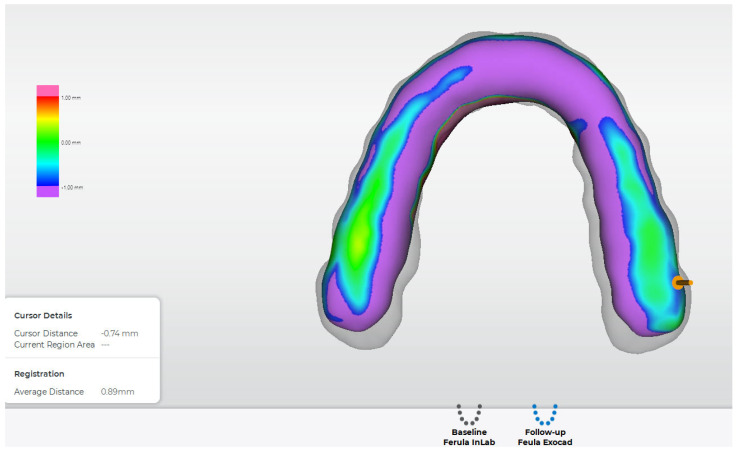
OraCheck 5.0 colour-coded deviation map of the ExoCAD splint vs. InLab superimposition.

**Figure 15:**
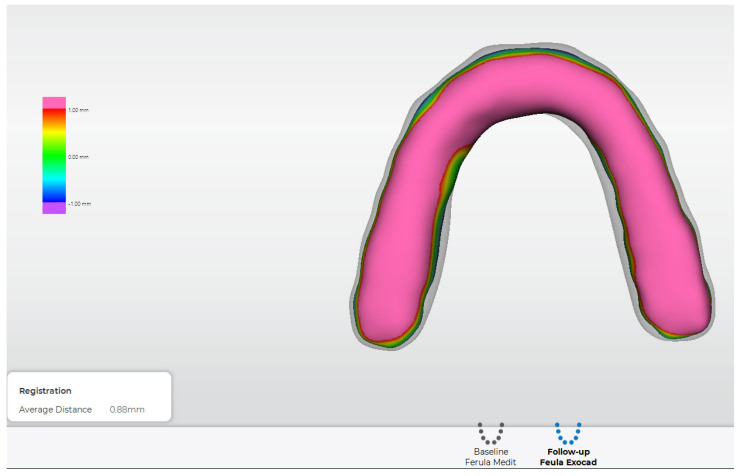
OraCheck 5.0 colour-coded deviation map of the ExoCAD splint vs. Medit Link superimposition.

**Figure 16:**
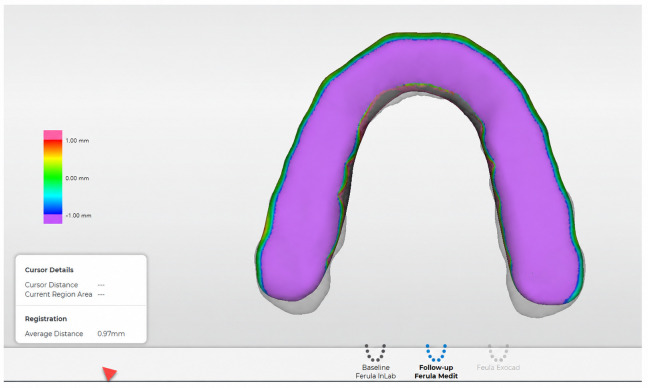
OraCheck 5.0 colour-coded deviation map of the InLab splint vs. Medit Link superimposition.

Three-dimensional best-fit superimposition (OraCheck 5.0) was performed on representative splint pairs to provide a descriptive assessment of geometric agreement between each CAD-designed splint and the control splint. Mean point-to-surface distances are summarized in Table 9. When each representative CAD-designed splint was compared with the control splint, InLab presented the smallest mean surface distance (0.36 mm), followed by ExoCAD (0.70 mm) and Medit Link (0.83 mm). Comparisons between representative CAD-system pairs yielded larger deviations: ExoCAD vs. Medit Link, 0.88 mm; ExoCAD vs. InLab, 0.89 mm; and Medit Link vs. InLab, 0.97 mm. Color-coded deviation maps are shown in Figure 12, Figure 13, Figure 14, Figure 15 and Figure 16.

The distribution of point-to-surface deviations across predefined intervals (Table 10) provides additional spatial information on geometric agreement. The InLab vs. Control comparison showed the highest proportion of points within 0.1 mm (59%) and the lowest discarded area (0%). The Medit Link vs. Control comparison showed an intermediate behaviour (43% within 0.1 mm; 9% discarded area), and the ExoCAD vs. Control comparison showed 33% of points within 0.1 mm and 23% of points exceeding 1.0 mm. Comparisons between CAD systems consistently displayed higher proportions of deviations >1.0 mm (19–36%).

## 4. Discussion

The present in vitro study evaluated whether different CAD software systems influence the accuracy of vertical dimension increase in digitally designed occlusal splints, and whether the resulting geometric differences affect biomechanical behavior under simulated occlusal loading. The null hypothesis was rejected, as differences were observed among the evaluated systems in linear vertical dimension, three-dimensional surface superimposition, and biomechanical response. Although digital workflows allow controlled modification of the vertical dimension of occlusion, this process cannot be considered inherently reliable: the variability between CAD systems shows that digital articulation does not fully replicate clinical conditions and should not replace conventional reference methods such as anterior jig-based determination [16,17]. Under identical input conditions, the three CAD systems produced different vertical dimension outcomes—Medit Link closest to the reference, InLab markedly overestimating it, and ExoCAD showing intermediate values—confirming that CAD software platforms are not interchangeable when modifying vertical dimension.

From a metrological perspective, the simultaneous evaluation of trueness and precision recommended by ISO 5725 [23] provides complementary information that cannot be replaced by a single global accuracy index [26]. The one-way ANOVA showed statistically significant differences between the four groups for both sides (*p* < 0.001) with extremely large effect sizes (η^2^ = 0.995 right; η^2^ = 0.987 left); group membership accounted for more than 98% of the total variance. The combined use of η^2^, ω^2^, and Cohen’s d, encouraged by current biomedical guidelines [25], reinforced the robustness of the conclusions: ω^2^ (less biased than η^2^) yielded virtually identical estimates, and pairwise Cohen’s d values consistently exceeded the conventional threshold of 0.8 for a large effect [25], with the largest contrasts observed against the InLab workflow (|d| up to 32.5).

The Medit Link group yielded the smallest absolute linear deviation from the reference (0.120 mm, right; 0.300 mm, left), placing this workflow within the clinically acceptable threshold of approximately 100–300 µm proposed for short-span and full-arch digital workflows in prosthodontics [27,28]. The ExoCAD group showed intermediate trueness (1.679 mm right; 1.121 mm left), consistent with reports indicating that CAD/CAM workflow performance depends not only on the input STL but also on the post-acquisition mesh-processing pipeline, where automatic tessellation, simplification, and alignment routines introduce additional sources of error [29,30]. The InLab group showed the largest linear deviation (≈4.0 mm right; ≈3.9 mm left) and the largest pairwise effect sizes (|d| > 14, *p* < 0.001 in every comparison). A systematic bias of this magnitude is well above the trueness values typically reported for digital splint workflows and would not be acceptable for prosthodontic purposes if reproduced in vivo. Possible contributing factors include the algorithmic strategy used to rotate the mandibular STL around a virtual condylar axis, differences in the definition of incisal landmarks across software platforms, and the way each CAD system manages occlusal contact resolution at the augmented vertical dimension [22,31,32].

These findings are consistent with previous studies evaluating digital articulation systems. Lin et al. [31] showed that vertical dimension changes differ between mechanical and virtual articulation due to variations in rotational axis simulation, Martani [32] reported discrepancies in occlusal contact simulation among virtual articulators, and Šimunović et al. [33] highlighted the lack of standardization in digital mandibular modeling. From a workflow perspective, additive manufacturing did not appear to be the primary source of error: as reported by Popescu et al. [34], 3D printing technologies are capable of accurately reproducing complex geometries, and discrepancies appear to originate during the CAD design phase—where mesh processing, occlusal alignment, and virtual articulation algorithms introduce clinically relevant deviations.

Beyond trueness, the precision (intra-group repeatability) of each workflow showed measurable differences between platforms, even though Levene’s test did not reach statistical significance (*p* > 0.05). Within-group SDs ranged from 0.087 to 0.132 mm on the right side and from 0.107 to 0.215 mm on the left side. The Control group showed the narrowest dispersion, as expected for direct measurement of a single physical reference. The InLab workflow consistently produced the largest dispersion, particularly on the left side (SD = 0.215 mm), in agreement with the observation that algorithmic re-articulation can introduce additional variability in the position of the mandibular STL [31,35]. The Medit Link and ExoCAD workflows showed a balanced performance in precision, comparable to values previously reported for short-span CAD/CAM splint workflows (SDs ≈ 0.10–0.15 mm) [29,36].

An important and counterintuitive observation emerged from the representative 3D superimposition analysis. While InLab showed the largest linear deviation in vertical dimension, the same system presented the smallest mean point-to-surface distance versus the control splint (0.36 mm) and the highest proportion of points within 0.1 mm (59%) among the representative comparisons. This apparent discrepancy may be interpreted within the metrological framework used in the present study: a linear caliper measurement captures a single one-dimensional value at a specific anatomical landmark (incisal zenith) and is therefore highly sensitive to directional bias along that axis, whereas a 3D best-fit superimposition averages distances across the entire occlusal and intaglio surfaces and is influenced by the overall geometry of the splint. Thus, the OraCheck findings suggest that the InLab discrepancy may have been mainly related to a directional bias affecting the incisal/vertical region, rather than to a generalized surface distortion of the entire splint. This interpretation is consistent with surface metrology principles, where best-fit alignment may partially compensate for localized deviations confined to limited portions of the surface [26,30].

Conversely, although Medit Link produced the linear measurements closest to the primary reference values, its representative global mean surface deviation versus the control splint was the highest of the three CAD systems (0.83 mm), with 9% of discarded area. This suggests that Medit Link reproduced the intended vertical dimension increase at the incisal landmark more closely, while showing larger distributed deviations across other regions of the splint surface.

These deviations may be related to software-dependent differences in mesh processing, smoothing, occlusal surface generation, or surface reconstruction; however, this explanation remains speculative and should be confirmed in future full-sample 3D analyses. ExoCAD showed an intermediate behavior in both linear deviation (1.68 mm right; 1.12 mm left) and representative 3D mean distance versus the control splint (0.70 mm).

Recent evidence supports the increasing role of fully digital workflows in occlusal splint fabrication, while also emphasizing that accuracy is influenced by multiple workflow-dependent factors, including CAD design strategy, manufacturing technology, printing orientation, material selection, and post-processing procedures [37,38,39]. Recent studies on 3D-printed occlusal splints have reported that trueness and precision may vary according to fabrication parameters and printing orientation, reinforcing that digitally fabricated splints should not be considered universally interchangeable [37,38]. Similarly, comparative studies of CAD/CAM splints have shown that manufacturing method, material behavior, positioning, and measurement strategy can influence the final fit and dimensional accuracy of the appliance [39]. These findings are consistent with the present study, in which all splints were generated from the same standardized dataset but showed different linear and surface-based outcomes depending on the CAD software used. The present results extend this concept by showing that variability may arise not only during manufacturing, but also during the CAD design stage when vertical dimension increase is digitally defined.

Comparisons between CAD systems showed higher proportions of deviations >1.0 mm (19–36%), suggesting that geometric differences among CAD platforms may be greater than those observed between some CAD-generated splints and the control splint. These findings reinforce the need for caution when considering CAD platforms as interchangeable for vertical dimension modification in occlusal splint design [29,36,40]. Therefore, the representative 3D superimposition analysis should be interpreted as complementary descriptive information regarding spatial deviation patterns, rather than as statistically generalizable evidence of differences among CAD systems.

The 0.1 mm cut-off used to interpret the OraCheck distribution is consistent with the clinically accepted tolerance for occlusal interfaces and intraoral scanning accuracy, which has been repeatedly reported in the range of ~50–100 µm for short-span anterior segments and full-arch comparisons in vitro [26,29,35,36]. Deviations below this threshold are generally considered indistinguishable from operator and articulation noise and lie within the functional adjustment range of clinically acceptable occlusal contacts; deviations above 0.5 mm, in contrast, are likely to require chairside correction. We therefore use 0.1 mm as a conservative pass/fail boundary rather than as a hard biological tolerance.

Clinically, these combined findings are highly relevant. Overestimation of vertical dimension may result in increased splint thickness, altered occlusal contacts, and the need for extensive chairside adjustments, in contrast with the efficiency advantages reported by Blasi et al. [41], who described reduced adjustment time when CAD-CAM splints achieve high accuracy. From the perspective of temporomandibular disorders, Somogyi et al. [42] emphasized that successful splint therapy depends on accurate transfer of mandibular position and controlled occlusal loading, while Albagieh et al. [43] highlighted that—despite workflow efficiency advantages—precision in vertical dimension modification remains a critical factor in treatment success. Software-dependent variations in occlusal contact interpretation [44] further reinforce the influence of CAD-specific factors on clinical outcomes. The representative 3D superimposition performed in the present study using OraCheck 5.0 [24] provided complementary descriptive information on the spatial distribution of surface deviations. The findings suggest that the linear deviation observed in the InLab workflow may have been mainly directional and localized in the incisal/vertical region, rather than reflecting a generalized distortion of the entire splint geometry. Conversely, Medit Link and ExoCAD showed more distributed surface deviations in the representative comparisons; however, these observations should be interpreted cautiously and should not be considered statistically generalizable evidence of differences in intaglio adaptation among CAD systems.

However, it should be noted that no universally accepted clinical threshold currently exists for digital vertical dimension modification in occlusal splint design. Therefore, the deviations observed in the present study should be interpreted as comparative in vitro findings rather than as direct indicators of clinical acceptability or failure. Their clinical relevance may depend on the intended therapeutic objective, splint design, occlusal adjustment protocol, patient adaptation, and follow-up stability.

From a biomechanical perspective, the finite element analysis suggested that CAD-derived geometric differences may influence both stress distribution and local deformation behavior under standardized parafunctional loading. The Medit Link geometry showed the highest equivalent von Mises stress values, whereas the ExoCAD and Medit Link geometries showed greater local deformation amplitudes in the premolar path analysis. These findings suggest that geometric accuracy in reproducing the intended vertical dimension does not necessarily imply a more favorable biomechanical response, highlighting the complexity of CAD-driven splint performance.

The path-based deformation analysis provided additional information regarding local deformation gradients across the splint thickness. By reporting ΔDef_path rather than isolated nodal values, the analysis captured the deformation amplitude between the most and least displaced regions of each predefined premolar path. This approach reduces the risk of overinterpreting single-point values and provides a more robust local descriptor of mechanical behavior.

The control geometry showed the smallest deformation ranges, whereas the CAD-designed geometries showed greater local deformation amplitudes. These differences may be associated with CAD-derived variations in thickness, surface morphology, and occlusal contact distribution, which can influence the local mechanical response of the splint even when all models are analyzed under identical material, contact, boundary, and loading conditions. This is consistent with previous FEA studies demonstrating that occlusal splint biomechanics are sensitive to local geometry, thickness, and material properties [12,13,14,15,45].

The maximum von Mises stress values remained below the flexural strength range generally reported for rigid post-cured methacrylate-based splint resins. Assuming a reference flexural strength of approximately 100 MPa, all evaluated geometries presented a factor of safety greater than 1 under the simulated static loading condition. However, this interpretation should be considered only as a structural reference under the selected assumptions. Fatigue, wear, intraoral aging, water sorption, cyclic parafunctional loading, saliva, and patient-specific neuromuscular dynamics were not included in the model. Therefore, the FEA results should be interpreted as comparative indicators of structural efficiency rather than as definitive predictors of long-term clinical performance.

The use of frictional contact between the splint and the dental structures represents a more realistic interface than a fully bonded condition, because occlusal splints are clinically retained by adaptation, friction, and undercut engagement rather than by adhesion. Nevertheless, the contact model remains a simplification of the oral environment. The applied 800 N load was selected to represent a severe parafunctional scenario and to magnify differences among the CAD-derived geometries under standardized worst-case conditions.

This study has several limitations that should be considered when interpreting the present findings. *(i)* It was conducted using a single standardised maxillary digital model derived from one articulator-mounted master cast; consequently, the results cannot be directly extrapolated to clinical scenarios involving severe malocclusion, partial edentulism, or anatomically atypical arches, and the work should be interpreted as a methodological demonstration rather than a definitive cross-population comparison. *(ii)* The in vitro design does not replicate the complexity of the oral environment, including saliva, occlusal function, parafunctional loading, and individual biological variability. *(iii)* The full digital workflow was executed by a single experienced operator using a single intraoral scanner configuration, a single splint material (Night Guard Firm 2) and a single additive manufacturing system (SprintRay Pro, with SprintRay ProWash/Procure post-processing); inter-operator, inter-scanner, inter-resin and inter-printer variability were therefore *not* assessed and should be addressed in future multi-centre studies. *(iv)* The finite element model assumed linear-elastic isotropic material behaviour under a static, geometrically standardised load; cyclic, viscoelastic, time-dependent and frictional contact phenomena were not simulated, which limits the direct extrapolation of the biomechanical outputs to long-term clinical performance. *(v)* The OraCheck 5.0 superimposition was performed on representative samples per CAD group rather than on all 20 splints per group; the surface-based outputs are therefore descriptive indicators of geometric agreement and should not be interpreted as inferential estimators (no parametric or bootstrap CIs were computed for the 3D analysis, *n* = 1 per comparison). Future studies should expand the surface-based comparison to the entire sample, incorporate signed-distance and volumetric analyses, and report inferential statistics with bootstrap or permutation-based confidence intervals [30,37]. *(vi)* Although the in vitro framework allowed strict control of confounding variables, clinical (in vivo) validation against patient-derived intraoral scans and longitudinal occlusal monitoring remains an essential next step. Finally, the results may vary depending on software versions and material properties, so periodic re-validation of CAD workflows is recommended.

The present study did not assess reliability in the strict metrological sense, as no intra-operator or inter-operator repeated-measures protocol, test–retest analysis, or intraclass correlation coefficient was performed. Therefore, the term reproducibility refers only to the dispersion of repeated design outputs generated under standardized conditions within each CAD workflow.

A further limitation is that the twenty splints generated per CAD system were repeated design outputs derived from the same STL dataset and created by the same operator under standardized conditions. Therefore, they should not be interpreted as fully independent biological specimens. This design was selected to isolate the effect of the CAD software workflow; however, it does not account for anatomical variability among patients, inter-operator variability, or test–retest reliability across different users.

Within the limitations of this study, the four CAD-based workflows produced statistically and clinically different linear measurements with respect to the master cast baseline. Medit Link offered the highest linear trueness, comparable to the reference, while InLab introduced a directional bias well above the clinically accepted threshold for the linear vertical dimension; however, the same InLab workflow produced the lowest 3D mean surface deviation vs. control. These findings reinforce the recommendation that the accuracy of CAD-based occlusal splint design should be evaluated both linearly (at clinically meaningful landmarks) and globally (using best-fit STL superimposition), as a single index does not capture the full picture of geometric performance [23,26,36,37]. This study challenges the assumption that digital workflows can independently determine clinically valid mandibular positions without verification, and supports the need for larger sample sizes, dynamic loading simulations, different splint materials, and clinical validation in future research.

Considering the in vitro nature of the present work, future research should pursue: (i) full-sample three-dimensional superimposition with inferential statistics, including signed-distance and volumetric analyses with bootstrap confidence intervals; (ii) multi-operator, multi-scanner, multi-printer, and multi-material designs to quantify clinically relevant sources of variability; (iii) dynamic and viscoelastic finite element simulations under cyclic loading; and (iv) prospective in vivo validation of the present CAD workflows in patients receiving occlusal splint therapy with controlled vertical dimension modification, including longitudinal follow-up of occlusal stability, chairside adjustment time, and patient-reported outcomes.

## 5. Conclusions

Within the limitations of this in vitro study, CAD software selection influenced the accuracy and reproducibility of vertical dimension increase in digitally designed occlusal splints. The null hypothesis was rejected. Medit Link produced the linear measurements closest to the primary reference values, whereas InLab showed the greatest linear overestimation and ExoCAD presented intermediate deviations.

Representative three-dimensional superimposition provided complementary descriptive information, showing that linear and surface-based metrics are not interchangeable. The FEA results suggested that CAD-derived geometric differences may influence stress distribution and local deformation behavior under standardized static loading; however, these findings should be interpreted as comparative biomechanical indicators rather than definitive predictors of clinical performance.

Careful verification of CAD design parameters against a conventional clinical reference is advisable before manufacturing occlusal splints involving vertical dimension modification.

## Figures and Tables

**Table 1:** Linear measurements (mm) obtained in baseline conditions.

Position	Right (mm)	Left (mm)
Baseline VDO position	16.50	15.53
Increased VDO position	22.68	21.10

**Table 2:** Descriptive statistics of right and left measurements (mm) per group, with 95% CI for the mean and trueness expressed as deviation from the baseline reference.

Side	Group	*n*	Mean ± SD (mm)	95% CI (mm)	Min − Max	Trueness (mm)	|Trueness| (mm)
Right	Control	20	23.50 ± 0.09	23.46 − 23.54	23.34 − 23.66	+0.818	0.818
Right	ExoCAD	20	24.36 ± 0.11	24.31 − 24.41	24.13 − 24.50	+1.679	1.679
Right	InLab	20	26.70 ± 0.13	26.64 − 26.76	26.43 − 26.99	+4.020	4.020
Right	Medit Link	20	22.80 ± 0.11	22.75 − 22.85	22.66 − 23.03	+0.120	0.120
Left	Control	20	22.50 ± 0.11	22.45 − 22.55	22.31 − 22.73	+1.399	1.399
Left	ExoCAD	20	22.22 ± 0.18	22.14 − 22.30	21.81 − 22.48	+1.121	1.121
Left	InLab	20	25.00 ± 0.22	24.90 − 25.10	24.59 − 25.51	+3.901	3.901
Left	Medit Link	20	21.40 ± 0.11	21.35 − 21.45	21.20 − 21.62	+0.300	0.300

**Table 3:** Shapiro–Wilk normality test for each group and side (*n* = 20).

Side	Group	*n*	W	*p*-Value	Distribution
Right	Control	20	0.976	0.869	Normal
Right	ExoCAD	20	0.946	0.305	Normal
Right	InLab	20	0.980	0.935	Normal
Right	Medit Link	20	0.934	0.181	Normal
Left	Control	20	0.970	0.757	Normal
Left	ExoCAD	20	0.959	0.517	Normal
Left	InLab	20	0.952	0.398	Normal
Left	Medit Link	20	0.922	0.107	Normal

**Table 4:** Results of the one-way ANOVA for between-group comparison of measurements per side, including effect-size estimates.

Side	Test	F	df_1_	df_2_	*p*-Value	η^2^	ω^2^
Right	One-way ANOVA	4821.82	3	76	<0.001	0.995	0.994
Left	One-way ANOVA	1904.68	3	76	<0.001	0.987	0.986

**Table 5:** Pairwise comparisons (Tukey’s HSD) between groups for each side. Mean difference (A − B) is reported with the 95% CI; q is the studentized range statistic; Cohen’s d is reported as the effect size for each pairwise contrast.

Side	Group A	Group B	Mean Diff (mm)	95% CI (mm)	q	*p*-Adj	Cohen’s d
Right	Control	ExoCAD	−0.862	[−0.953, −0.771]	35.25	<0.001	−8.82
Right	Control	InLab	−3.203	[−3.294, −3.111]	130.95	<0.001	−28.74
Right	Control	Medit Link	+0.698	[+0.606, +0.789]	28.52	<0.001	+7.17
Right	ExoCAD	InLab	−2.341	[−2.432, −2.249]	95.70	<0.001	−19.46
Right	ExoCAD	Medit Link	+1.560	[+1.468, +1.651]	63.77	<0.001	+14.54
Right	InLab	Medit Link	+3.900	[+3.809, +3.991]	159.47	<0.001	+32.54
Left	Control	ExoCAD	+0.278	[+0.145, +0.410]	7.80	<0.001	+1.89
Left	Control	InLab	−2.502	[−2.634, −2.370]	70.36	<0.001	−14.72
Left	Control	Medit Link	+1.098	[+0.966, +1.230]	30.88	<0.001	+10.18
Left	ExoCAD	InLab	−2.780	[−2.912, −2.647]	78.16	<0.001	−14.08
Left	ExoCAD	Medit Link	+0.821	[+0.688, +0.953]	23.07	<0.001	+5.57
Left	InLab	Medit Link	+3.600	[+3.468, +3.732]	101.24	<0.001	+21.14

**Table 6:** Equivalent von Mises stress results. Minimum, stress range, and maximum values are reported in MPa.

Geometry	Min	Stress Range, Max − Min (MPa)	Max
Control	0.00041	19.6056	19.606
InLab	0.03994	31.9611	32.001
ExoCAD	0.00576	32.8822	32.888
Medit Link	0.17144	40.4706	40.642

**Table 7:** Total deformation results. Minimum, deformation range, and maximum values are reported in mm.

Geometry	Min	Deformation Range, Max − Min (mm)	Max
Control	8.76 × 10^−7^	0.0128881	0.0128890
InLab	1.01 × 10^−4^	0.0514515	0.0515530
ExoCAD	0.000000	0.0594630	0.0594630
Medit Link	0.0041450	0.0372100	0.0413550

**Table 8:** Deformation along predefined paths in the premolar regions. Minimum deformation, path deformation range, and maximum deformation are reported in mm. Note: ΔDef_path represents the deformation range along each predefined path and was calculated as Def_max − Def_min. It should not be interpreted as an average or an absolute total deformation value.

Geometry	Path	Def_min (mm)	∆ Def_path (mm)	Def_max (mm)
Control	Right	0.000004	0.000195	0.000199
Control	Left	0.000009	0.000188	0.000197
InLab	Right	0.002103	0.019537	0.021640
InLab	Left	0.001704	0.011030	0.012734
ExoCAD	Right	0.412270	0.019050	0.431320
ExoCAD	Left	0.173700	0.016652	0.189560
Medit Link	Right	0.000068	0.022374	0.022442
Medit Link	Left	0.004145	0.015860	0.041355

**Table 9:** Mean point-to-surface distance (mm) obtained by 3D superimposition (OraCheck 5.0) for the six pairwise comparisons.

Pairwise Comparison	Mean Distance (mm)
ExoCAD vs. Control	0.70
Medit Link vs. Control	0.83
InLab vs. Control	0.36
ExoCAD vs. Medit Link	0.88
ExoCAD vs. InLab	0.89
Medit Link vs. InLab	0.97

**Table 10:** Distribution of point-to-surface deviations and discarded area (%) per pairwise STL superimposition (OraCheck 5.0).

Pairwise Comparison	0–0.1 mm	0.1–0.5 mm	0.5–1.0 mm	>1.0 mm	Discarded Area
ExoCAD vs. Control	33%	26%	17%	23%	1%
Medit Link vs. Control	43%	22%	7%	18%	9%
InLab vs. Control	59%	17%	9%	15%	0%
ExoCAD vs. Medit Link	38%	12%	15%	36%	0%
ExoCAD vs. InLab	31%	18%	19%	31%	1%
Medit Link vs. InLab	43%	21%	7%	19%	9%

## Data Availability

The datasets generated and analysed during the current study are available from the corresponding author on reasonable request.
